# After COVID-19 We Will Need a New Research System. We Need To Start Planning Now

**DOI:** 10.1128/mBio.03251-20

**Published:** 2020-11-20

**Authors:** Stefano Bertuzzi, Victor J. DiRita

**Affiliations:** a American Society for Microbiology, Washington, DC, USA; b Department of Microbiology & Molecular Genetics, Michigan State University, East Lansing, Michigan, USA

## EDITORIAL

The world after COVID-19 will be significantly different than the one we thought we knew so well only months ago. Only one thing is clear to us about the post-COVID-19 future, the scientific research system will change and we must start planning for that change now.

COVID-19 has revealed a biomedical research enterprise at once powerful and yet remarkably vulnerable. On one hand, drawing from a bedrock of coronavirus research, in practically no time the enterprise has registered well over 300 interventional clinical trials and over 100 vaccine trials. Thanks to vaccine platform technology, we have seen the fastest first in human test for a vaccine ever and preliminary results from two Phase III clinical trials, concluded in record time, indicate extremely encouraging results. On the other, this novel, highly contagious virus has made a mockery of advanced scientific countries around the world with its unpredictability and atypical outcomes in different human populations. So far, the most effective strategy has been to wear a mask, “stay home and wash your hands frequently.” This is essentially the same advice our grandmothers, who survived the 1918 pandemic flu, used to give us all the time.

However this pandemic plays out, we think that the scientific ecology that once supported the “old” research system will be vastly different afterward. We do not mean merely the scientific knowledge we gain about pandemics or even the field of microbiology, but the whole niche in our society where basic and applied research has flourished for decades. Funding, education, institutional stability, government policy, international cooperation, demographics, information flow—all will be changed. But how do we plan for this?

As the Chief Executive and the President of the American Society for Microbiology (ASM) during this crisis, we have been working nonstop to channel the efforts of ASM members and the society’s resources to counter the immediate threat of COVID-19 by deploying our special skills, knowledge, and perspectives. These efforts have also been recognized by the American Society of Association Executives (ASAE) awarding ASM the prestigious Golden Circle Award for impact on society at large, of which we are justifiably proud. But we need to pause for a second and look ahead. We try to imagine where COVID-19 will leave scientific research in the months and years ahead. Oddly enough in looking ahead, we find ourselves looking back as this year we celebrate the 75th anniversary of Vannevar Bush’s landmark report, *Science*, *The Endless Frontier* (https://nsf.gov/od/lpa/nsf50/vbush1945.htm). We now believe that an updated version of his “Frontier” report offers the best way forward for research science after COVID-19.

The name Vannevar Bush doesn’t spark much recognition these days unless you are an MIT alum who took classes in Building 13, the large campus building renamed for him in 1965, or if you wondered whether he was related to the presidential Bush family tree (No). Yet Vannevar Bush was probably the most influential American scientist of the 20th century. He is remembered not so much for his science (although as an electrical engineer, V. Bush laid the groundwork for what became the computer revolution) as for his scientific leadership. In World War Two (WWII), Vannevar Bush mobilized American scientists (as well as foreign scientific refugees including Albert Einstein) behind the war effort through his Office of Scientific Research and Development. In late 1944 while fighting was still raging in Europe and the Pacific, President Franklin Roosevelt sent him a letter, asking for his advice on four points concerning postwar American science. In his response, Bush laid the foundations of modern scientific research. (Roosevelt, who died in April 1945, did not live to read Bush’s July 1945 report.).

Vannevar Bush’s vision was this: American research science, particularly in physics and biology, was key to national security and the public good. Federally supported investigators would have freedom of inquiry working in both public and private research institutions. Except for top military secrets, results would be published and international cooperation encouraged. Science education, from high school to postgraduate levels, was to be accessible to all with scientific talent, regardless of family background or gender. Short term, the Vannevar Bush report led to the creation of the National Science Foundation and the American research university. Long term, it led to the incredible system of innovation that the U.S. has enjoyed for the past 75 years—a partnership between federal government and universities that fostered phenomenal research, innovation, and exploration. It worked out very well for the U.S. and for the rest of the world.

Seventy-five years is an epoch in science. Even before COVID-19, the world of science and society had become incredibly complex and diverse. Yet, the framework of the four points laid out by Vannevar Bush gives us a roadmap to reboot the science endeavor of the future, especially in light of the seismic faults exposed by the COVID-19 pandemic.

**FIG 1:**
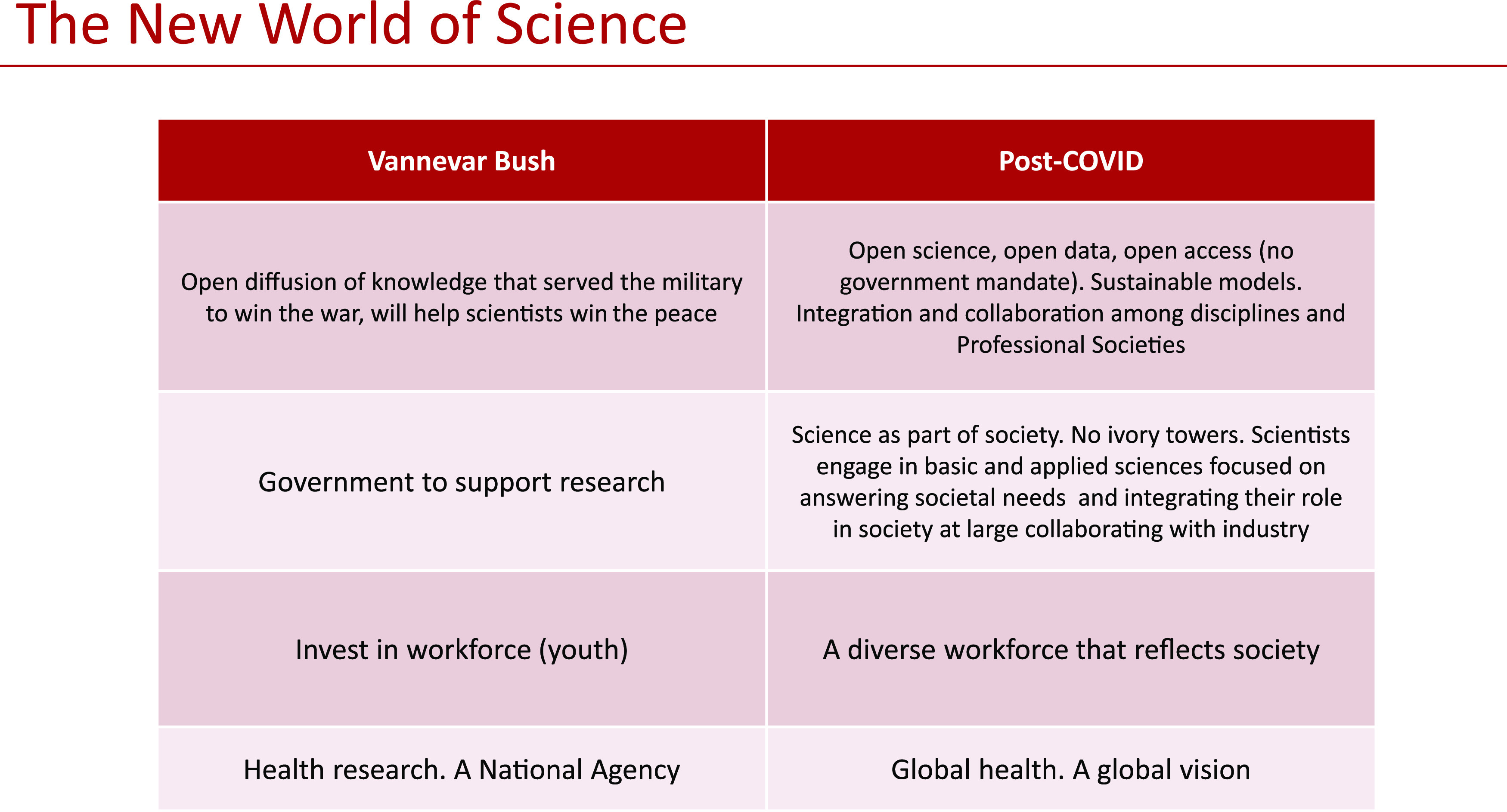
The new world of science. Vannevar Bush’s vision of science after WWII compared with our vision of science after COVID-19.

Here is our attempt to flesh out an update on Vannevar Bush’s four main points and describe a post-COVID-19 scientific landscape (Fig. 1):

One—Science needs to be open. Secrecy in wartime was necessary, Bush said, but after 1945, America needed to “lift the lid” on nearly all the new science generated by the war effort such as the development of penicillin. He urged open publication of federally funded research and full participation by American scientists in international conferences and exchanges. We would take this one step further—science must also be opened to the American public. We need to recognize that science must have the trust of society and that much of the recent public rejection of scientific credibility stems from the isolation of scientists behind jargon or institutional walls. If there is a bright side to COVID-19, it is that working scientists are active in public discussion again. The pandemic has unleashed a flood of raw or half-baked data, while at the same time preprints and rapid publication processes are an asset. Real scientists are being sought out to explain how the scientific method works, how data are evaluated, and why science changes its mind. From now on, scientists need to see themselves as integral to society and take on their responsibility to engage with society at large. They need to champion evidence-based science as a force in decision making and public sentiment. Importantly, they need to be clear about what is known and what remains to be discovered. The onus is on scientists to engage the public, become visible, and speak clearly. Talking only to each other in ivory towers is no longer an option. Science must also be open—aggressively so—to other scientists. Peer review of data obviously remains essential, but the COVID-19 pandemic has illuminated how preprint servers like medRxiv can ensure widespread, rapid dissemination of new knowledge.

Two—We would double down on Vannevar Bush’s inclusion of basic biology and medical research as fundamental to national security. This link was a surprising cornerstone of Vannevar Bush’s plan. Before WWII, research was the province of big medical schools and a handful of privately endowed institutions. Bush made basic biology an ongoing national priority. Today while we are scrambling to come up with a vaccine to respond to a completely new virus, we should not forget that without years of basic research on coronaviruses, immunology, molecular genetics, and -omics technologies, vaccine makers would be blundering about in the dark. Critics have long grumbled about the cost of “pointless” basic research. Consider the costs of basic ignorance. This is another area where clarity from scientists in speaking to the public is essential: our search for new knowledge in biology rarely leads down a direct, predictable path from discovery to application. Producing the projected COVID-19 vaccine will also test the limits of cooperation between research science and industry to deliver a safe and effective vaccine in sufficient quantity and at manageable cost. In post-COVID-19 science, this link must be forged more tightly and yet more flexibly to protect future populations.

Three—We need better science education and training for a more diverse workforce. WWII changed American attitudes about many things, including gender. American women did many things during the war that women were never supposed to do, from flying military aircraft to welding ships to serving in frontline medical units. From labs to “calculating” rooms, women had a huge impact on wartime science that left Bush with no doubts about the make-up of his postwar scientific workforce. “We must have plenty of men and women trained in science,” he wrote in 1945. He also wrote, “Studies show that there are talented individuals in every part of the population.” We would make this more explicit: the research science workforce must become more open to all talents. The scientific workforce must reflect the diversity at all levels of today’s society. The post-COVID-19 science reboot will take full advantage of the enormous demographic and intellectual diversity in our society today to forge new approaches and solutions. This is not just the right thing to do in the name of social justice—it is that—but doing so will also give our new science its best chance to solve complex problems. The more diversity we foster, the greater our chances to go faster and further.

Four—Vannevar Bush offered a national vision for science. He understood the critical gift that European refugee scientists brought to the U.S. war effort, and he stressed the need for international science in peacetime, but “Frontiers” was a report to an American president. In the post-COVID-19 science world, we need to go wide, meaning worldwide. As our current state makes clear, microbes know no borders. There is no safety in scientific isolationism or biomedical ultranationalism. International planning and coordination will become even more essential in the future as COVID-19 is unlikely to be the last candidate for a global pandemic. In the post-COVID-19 world, we will need a scientific construct of open science, open data, and open access. This needs to be built together, not ideologically, or by top-down governmental mandates, but organically and thoughtfully by scientists themselves to ensure sustainability of the research endeavor. And we need to reconsider how we incentivize research progress. When confronting large problems—pandemics, antimicrobial resistance, climate change—large groups with diverse talent and expertise trained on the challenge from different perspectives can make progress much faster than individual laboratories jealously guarding their data from perceived competitors. The curiosity-driven individual grantee should not be forsaken, but our research universities have to find ways to foster and recognize large research teams. This raises new opportunities in how we train scientists, and how we reward them. Building teams should cross national frontiers as well: the future needs bioscience at a global level. We need to develop a system of scientific leadership where the U.S. can lead with other countries and continents on a globally coordinated level. Just like we have the United Nations, the World Bank, and the International Monetary Fund safeguarding planetary economic well-being, we will need a United Nations of Science or a Global Institute of Health to safeguard the hope that comes from science.

The report by Vannevar Bush started a golden era of U.S. science. For the new post-COVID-19 research science effort, we need to expand and modernize the rich ecosystem that has evolved between industry and science. This new construct will need to reflect not the science of 75 years ago, but the science of tomorrow, where scientific disciplines will intertwine and scientists and their scientific societies will no longer be caught up in turf battles but rather focus on what we can do together.

The COVID-19 pandemic is teaching us painful lessons. As scientists, policy experts, and politicians, let’s not waste the opportunity to reorient research toward new frontiers, truly endless and without borders.

